# Combined Anterolateral and Posterior Approach in Total Hip Arthroplasty for Chronic Post-traumatic Hip Dislocation: A Case Report

**DOI:** 10.7759/cureus.61558

**Published:** 2024-06-03

**Authors:** Issei Senga, Hisatoshi Ishikura, Naoto Kaminaga, Masashi Sato, Takeyuki Tanaka, Sakae Tanaka

**Affiliations:** 1 Department of Orthopedic Surgery, Saitama Red Cross Hospital, Saitama, JPN; 2 Department of Orthopedic Surgery, The University of Tokyo, Tokyo, JPN

**Keywords:** chronic dislocation, combined approach, dual mobility total hip arthroplasty, total hip arthroplasty (tha), traumatic posterior hip dislocation, hips

## Abstract

Hip dislocation is rare, and it typically results from high-energy trauma such as traffic accidents. Its management involves prompt reduction of the dislocated hip to minimize the risk of subsequent femoral head necrosis. Consequently, cases of chronic hip dislocation are extremely rare. This report presents a case of a 33-year-old male with chronic posterior hip dislocation due to a traffic accident 13 years ago. The left femoral head was completely dislocated posteriorly from the acetabulum, forming a false acetabulum with an arthritic change. The patient experienced difficulty walking and performing daily activities due to pain. We performed a total hip arthroplasty (THA) using a combined anterolateral and posterior approach. The outcome was favorable, with no complications during the two-year follow-up period. THA using a combined anterolateral and posterior approach is a valuable option for patients with chronic post-traumatic hip dislocation because it offers the advantages of optical visibility and the management of the adhered soft tissues.

## Introduction

Hip dislocation is rare, and it typically results from high-energy trauma such as motor vehicle accidents. It may also be associated with acetabular, femoral head, or proximal femoral fractures, making its treatment challenging [1].

Its management involves prompt reduction of the dislocated hip to minimize the risk of subsequent femoral head necrosis [2,3]. Swift intervention for associated fractures is also fundamental. Consequently, cases of chronic hip dislocation are extremely rare.

Chronic hip dislocation has been defined in previous reports as a hip dislocation persisting for more than six weeks after onset [4]. Open reduction and preoperative traction are considered the most common and accessible treatments. However, there are no reports on cases persisting for more than ten years and developing osteoarthritic changes.

This report presents a case of chronic hip dislocation that remained untreated for 13 years following a traffic accident. We performed a total hip arthroplasty (THA) using a combined approach to achieve favorable outcomes. This case highlights the successful use of the combined anterolateral and posterior approaches in managing chronic hip dislocation and presents a unique contribution to the existing literature.

## Case presentation

A 33-year-old male (height, 165 cm; weight, 62 kg; body mass index, 22.7 kg/m^2^) from Guinea presented with a 13-year medical history that started when he was 20 years old. At that time, he had a dislocated and fractured left hip joint and fractures of the left tibia and fibula following a traffic accident in his home country. The hip dislocation persisted after approximately 1 month of traction for the left lower limb. Efforts to seek medical attention at a comprehensive hospital in Guinea proved futile as the technical feasibility of the surgery was deemed impossible. Inquiries were made in neighboring countries, but pursuing medical interventions was abandoned because of exorbitant treatment costs. Subsequently, the patient continued to experience persistent pain in the left hip joint, accompanied by walking difficulties, as he managed to navigate through his daily activities and life in general. After a decade, he applied for refugee status and arrived in our country. Upon employment in our country, he gained access to medical care through the health insurance system, which made surgical interventions possible. The patient was referred to our institution for consultation. Notably, no other significant medical history was reported besides trauma.

Upon presentation, the patient used a single crutch or walker and exhibited a Trendelenburg gait. There was a significant leg-length discrepancy, and the entire left lower limb exhibited marked adduction and internal rotation, resulting in a toe-to-ground position of the left foot. The left hip had a limited range of motion, with flexion and abduction restricted to 80° and 20°, respectively. In addition to walking impairment and decreased activities of daily living, pain was also observed at rest. There were no neurological abnormalities such as paralysis or numbness in this patient.

**Figure 1:**
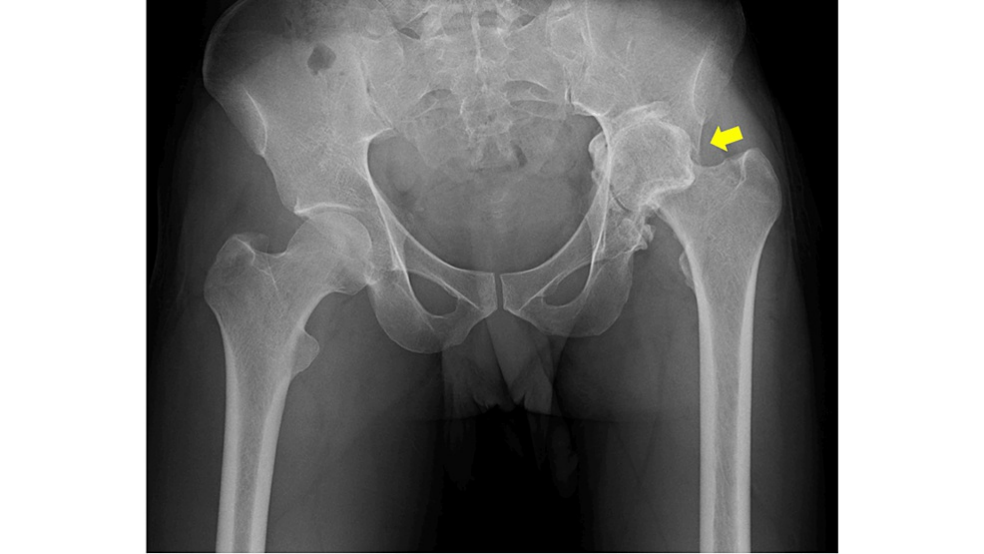
An anteroposterior plain radiograph of both hips at the initial visit. The left hip (arrow) exhibits upward displacement and central migration, resulting in a significant leg length discrepancy.

**Figure 2:**
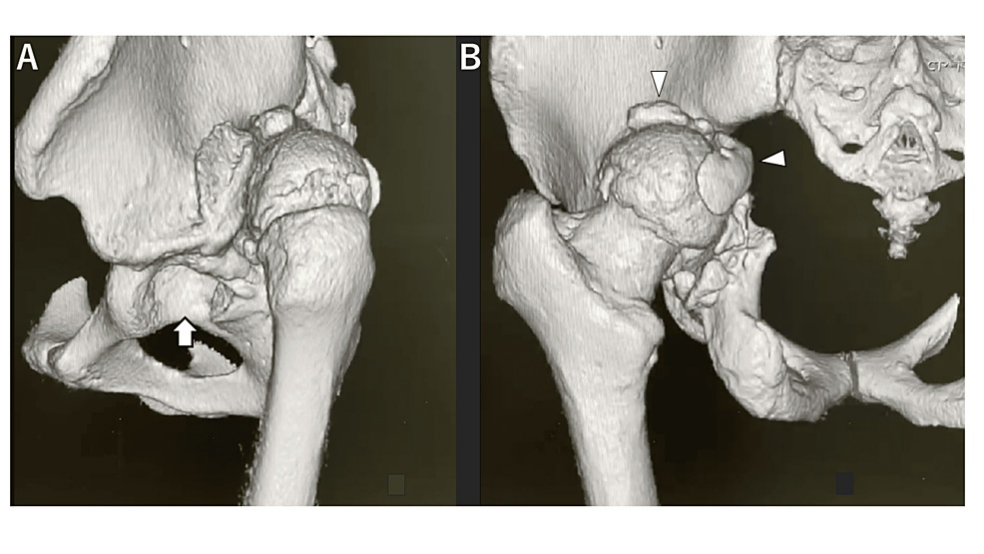
Three-dimensional reconstruction from plain CT scans at the initial visit. Three-dimensional reconstruction from plain CT scans, depicting views from the left side (A) and posteriorly (B). The left hip joint dislocates posteriorly, with the evident presence of the original acetabulum (A, arrow). Posterior to the original acetabulum, a false acetabulum is formed, and resulting bone spurs partially cover the femoral head (B, arrowhead).

Plain radiography revealed findings suggestive of central migration, with an approximate 3 cm leg-length discrepancy (Figure 1). Further examination using plain computed tomography (CT) indicated that the left femoral head was completely dislocated posteriorly from the acetabulum, forming a false acetabulum accompanied by an arthritic change (Figure 2).

**Figure 3:**
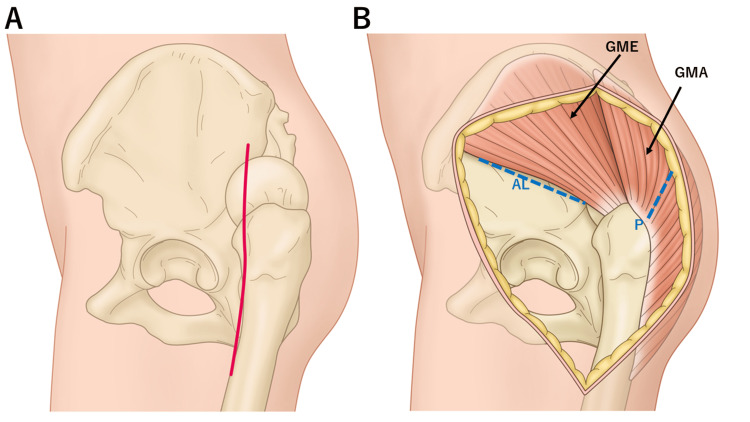
An illustration depicting the skin incision and the surgical entry paths. (A) An illustration depicting the incision sites on the skin and fascia positioned along the anterior edge of the femur. (B) An illustration depicting the surgical entry paths for the combined approach. The anterolateral approach is anterior to the gluteus medius, whereas the posterior approach involves splitting the gluteus maximus. Image credit: Hisatoshi Ishikura. GME, gluteus medius; GMA, gluteus maximus; AL, anterolateral; P, posterio

**Figure 4:**
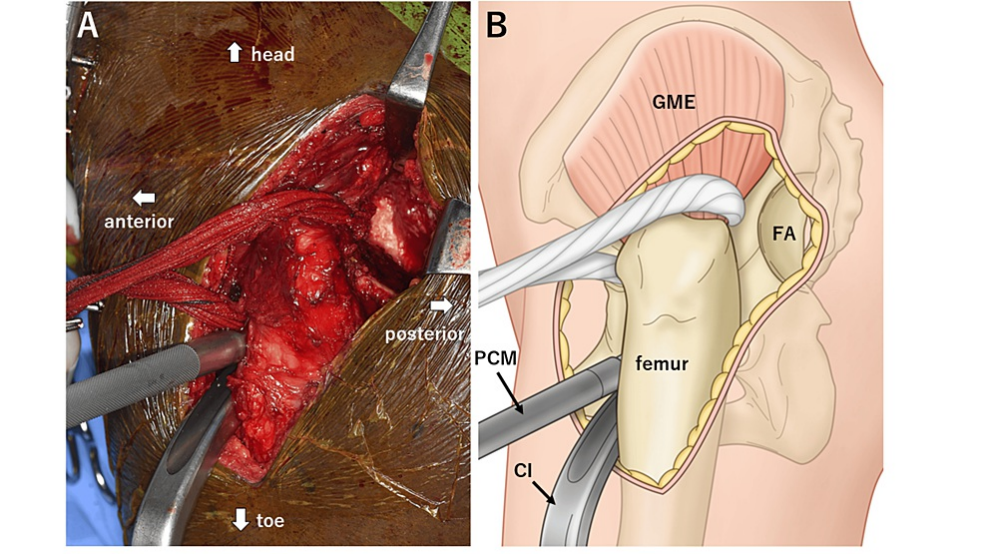
A picture and an illustration of the intraoperative field during total hip arthroplasty. (A) Intraoperative photograph and (B) corresponding illustration. The cup connected to the impactor is inserted from the anterolateral aspect, with the false acetabulum visible in the posterior aspect. Image credit: Hisatoshi Ishikura. GME, gluteus medius; FA, false acetabulum; PCM, pusher for cup medialization; CI, cup impactor

**Figure 5:**
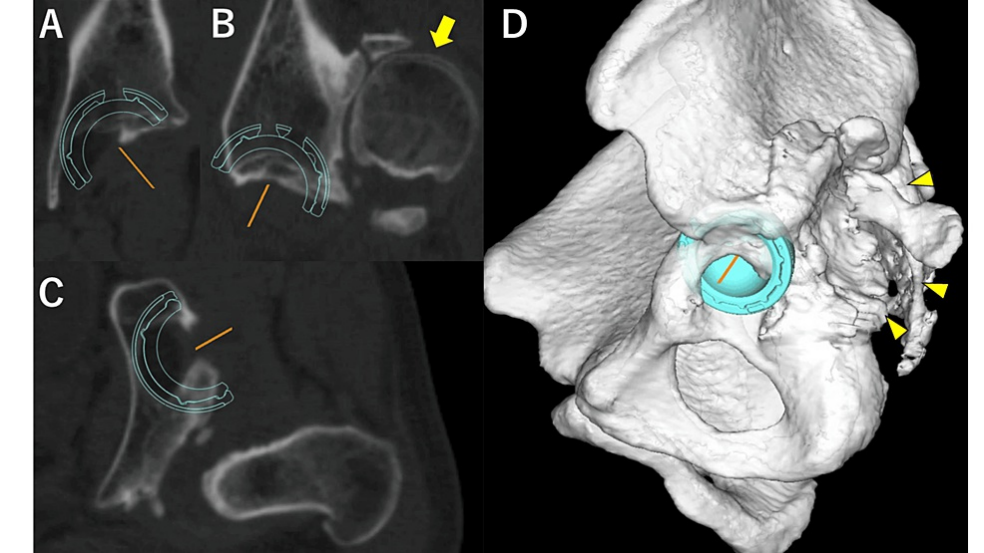
Preoperative planning using the computed tomography and 3D software to place a 42-mm cup. The images show (A) coronal, (B) sagittal, (C) axial, and (D) 3D reconstruction views. The dislocated femoral head (B, arrow) and the false acetabulum (D, arrowhead) are identifiable.

**Figure 6:**
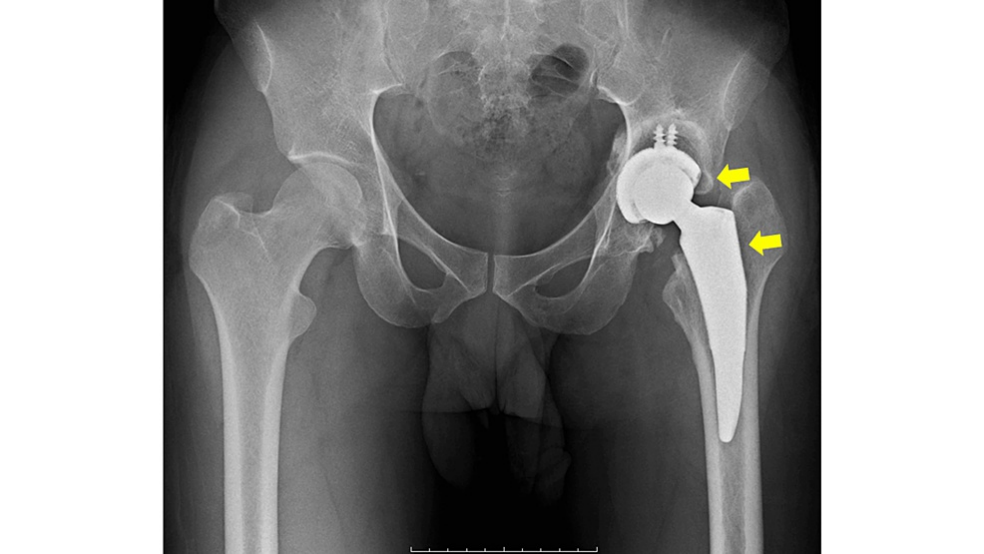
A postoperative anteroposterior plain radiograph of both hips. The implants (arrows) are well-placed as planned, and there is an improvement in leg length discrepancy compared to preoperative status.

THA was performed on this patient. Under general anesthesia, the patient was placed in the right lateral decubitus position. Initially, a single vertical incision was made slightly anterior to the center of the femur, and the femoral fascia was vertically incised (Figure 3A). Subsequently, the neck of the dislocated femur was osteotomized using a posterior approach. Osteotomy and femoral head extraction were carefully performed to prevent damage to the sciatic nerve located immediately behind the femoral head. An anterolateral approach was used for the acetabulum through the anterior aspect of the gluteus medius (Figures 3B-4). The implants used were meticulously planned preoperatively using the three-dimensional preoperative planning software (Zed Hip, LEXI, Tokyo, Japan) (Figure 5). A 42 mm G7 acetabular cup (Zimmer Biomet, Warsaw, IN) with dual mobility articulation was utilized, and the cup placement was facilitated using a CT-based navigation system (Stryker CT-based Hip Navigation System, Stryker-Leibinger, Freiberg, Germany). After cup placement, the femoral stem was inserted through a posterior approach, and Taperloc Microplasty (Zimmer Biomet) was performed. The surgery was performed in approximately 244 minutes, with a blood loss of 530 mL. Postoperative radiographs revealed successful implant placement based on the planned parameters, achieving an approximately 2 cm leg-length correction (Figure 6).

The patient began ambulation the following day. Rehabilitation was initiated, allowing weight bearing to be tolerated. There was an improved tendency for adduction and internal rotation of the lower limbs, and the subjective leg length discrepancy was alleviated. The patient was discharged on postoperative day 17, with independent ambulation using a T-cane. Subsequent follow-up revealed no complications, such as dislocation or infection. At the two-year postoperative mark, the patient demonstrated smooth ambulation without assistive devices, and the Harris Hip Score improved from 29.4 to 90.1.

## Discussion

THA is commonly performed using various approaches, such as the anterior, anterolateral, lateral, or posterior approaches [5]. In cases requiring a challenging exposure, such as ankylosed or highly dislocated hips due to dysplasia, a transtrochanteric or combined approach may be used. The transtrochanteric approach, which involves a greater trochanteric osteotomy, provides a good visual field. However, complications such as massive bleeding, heterotopic ossification, and nonunion have also been observed [6,7,8]. However, the combined approach achieves a favorable operative field without osteotomy. Furthermore, excellent outcomes were reported in a follow-up study spanning an average of 5.5 years for THA using the combined approach in 76 ankylosed hips [9]. Similarly, favorable results were reported in a follow-up study over an average of three years for THA using the combined approach in 71 highly dislocated hips (Crowe type IV) [10].

In this case, the posterior approach was advantageous for femoral neck osteotomy and stem manipulation due to the posterior hip dislocation. However, approaching the original acetabulum from the posterior direction was deemed impractical because of the longstanding posterior displacement and hip shortening. Consequently, the anterolateral approach was used for the acetabulum from the anterior aspect of the gluteus medius muscle. In this case, preoperative planning indicated that only a small cup could be placed (Figure 5). Therefore, a dual mobility cup was used, and CT-based navigation was employed to reduce the risk of dislocation [11]. Given this patient’s 13-year post-traumatic interval, extensive adhesions in the surrounding tissues made it difficult to pull the femur down. Therefore, the combined approach allowed for the release of tissues, including the anterior and posterior joint capsules, facilitating approximately 2 cm leg lengthening.

Complications such as nerve palsy have been reported to increase in cases of ankylosed or highly dislocated hips [12-14]. Especially, the sciatic nerve passed immediately behind the femoral head in the present case with chronic posterior dislocation. In addition, the femoral nerve ran closer than usual to the anterior edge of the acetabulum. Therefore, careful surgical maneuvers were performed, which resulted in the absence of postoperative sciatic or femoral nerve paralysis.

There was an improvement in the adduction and internal rotation of the left lower limb and an enhancement in the walking condition, leading to a highly satisfied patient. However, long-term follow-up is essential given that this case involved performing a THA in a young male.

Chronic traumatic hip dislocation is similar to Crowe grade IV high dislocation of the hip regarding shortening and contracture. However, it often differs significantly from the usual shape of the acetabulum after trauma, and there is a greater degree of adhesion to the surrounding tissues due to trauma. Therefore, addressing each case based on these considerations is essential. Nevertheless, even in chronic traumatic hip dislocation cases, a combined approach provided good visibility anteriorly and posteriorly, allowing for effective soft tissue management. Therefore, it proves to be a highly valuable technique.

## Conclusions

In this report, THA was performed using a combined anterolateral and posterior approach for chronic post-traumatic hip dislocation. This approach provided excellent visualization and allowed for effective management of atypical anatomy and soft tissue adhesions. Additionally, the short-term results were favorable without any complications.

This study is considered highly beneficial as it provides surgeons with a new option for the treatment of chronic post-traumatic hip dislocation.
